# Altered acetate metabolism and signaling in IgA nephropathy: an integrated gut microbiome and glomerular spatial transcriptome analysis

**DOI:** 10.3389/fimmu.2025.1665585

**Published:** 2026-01-14

**Authors:** Jung Hun Koh, Sehoon Park, Minji Kang, Ji In Park, Jangwook Lee, Hyunjeong Cho, Ji Eun Kim, Hoonsik Nam, Doyeon Kim, Minshu Li, Sunghyouk Park, Kyung Chul Moon, Hyun Je Kim, Yon Su Kim, Dong Ki Kim, Hajeong Lee

**Affiliations:** 1 Department of Internal Medicine, Seoul National University Hospital, Seoul, Republic of Korea; 2 Department of Internal Medicine, Seoul National University College of Medicine, Seoul, Republic of Korea; 3 Department of Biomedical Sciences, Seoul National University Graduate School, Seoul, Republic of Korea; 4 Department of Internal Medicine, Kangwon National University Hospital, Chuncheon, Republic of Korea; 5 Department of Internal Medicine, Dongguk University Ilsan Hospital, Ilsan, Republic of Korea; 6 Department of Internal Medicine, Chungbuk National University Hospital, Cheongju, Republic of Korea; 7 Department of Internal Medicine, Korea University Guro Hospital, Seoul, Republic of Korea; 8 College of Pharmacy, Natural Products Research Institute, Seoul National University, Seoul, Republic of Korea; 9 Department of Pathology, Seoul National University College of Medicine, Seoul, Republic of Korea

**Keywords:** glomerulonephritis, gut microbiome, IgA nephropathy, short-chain fatty acid, spatial transcriptomics

## Abstract

**Introduction:**

IgA nephropathy (IgAN) is the most common primary glomerulonephritis, and emerging evidence implicates the gut microbiome in its pathogenesis. Additional studies focusing on the molecular mechanisms linking gut microbial signals to intraglomerular changes are warranted.

**Methods:**

We performed 16S rRNA-based microbial profiling of fecal samples of 172 IgAN patients, 51 healthy controls, and other glomerular disease controls including 15 diabetic nephropathy, 35 minimal change disease, and 63 membranous nephropathy cases. Serum and fecal acetate levels were measured by liquid chromatography–mass spectrometry. Glomerular spatial transcriptomic profiling was performed with the GeoMx Digital Spatial Profiler. DESeq2 analysis was performed to identify differentially expressed genes, followed by gene ontology annotations.

**Results:**

Beta diversity differed significantly between IgAN and healthy controls (*p* = 0.001). While no single taxon showed consistent differences in abundance, the methanogenesis from acetate pathway was significantly enriched in IgAN, accompanied by an increased proportion of major acetate-producing gut microbial genera. Serum acetate levels were elevated in IgAN (*p* = 0.03), while fecal acetate levels were comparable to those in healthy controls. In glomerular transcriptomes, functional annotations of 1,227 upregulated and 1,078 downregulated genes in IgAN indicated decreased activities of G protein-coupled receptors, short-chain fatty acid transporters, and beta-1,3-galactosyltransferases.

**Discussion:**

IgAN is characterized by gut microbial enrichment in acetate metabolism and increased systemic acetate levels, along with altered intraglomerular expression of metabolic and signaling genes. These findings suggest a gut microbiome–glomerular signaling axis contributing to disease pathogenesis.

## 1 Introduction

Kidney diseases, spanning a broad spectrum of etiologies, are a growing health concern worldwide (1). Beyond traditional risk factors, gut microbial dysbiosis has been increasingly implicated in their pathophysiology (2, 3). Alterations in gut microbial composition and related metabolite profiles have been reported in chronic kidney disease (CKD) and in specific glomerular diseases (4–6), with associations to systemic consequences such as cardiorenal syndrome and mineral bone disorder (7, 8). The crosstalk between the gut microbiota and the kidney, dubbed the gut-kidney axis, involves multiple mediators including microbe-derived uremic toxins and other amino acid derivatives (9–11), whose causal associations to glomerular diseases were demonstrated by recent Mendelian randomization studies (11, 12). Together, a growing body of evidence indicates a key role of intestinal dysbiosis and metabolite imbalance across kidney diseases.

Immunoglobulin A (IgA) nephropathy (IgAN), the most prevalent primary glomerulonephritis worldwide, is closely connected to the gut microbiota in its immune-mediated pathophysiology. Central to its pathogenesis is the overproduction of galactose deficient-IgA1 (Gd-IgA1) and associated immune complexes (13, 14). Given the established role of IgA in mucosal immunity, host-microbiota interactions in mucosa-associated lymphoid tissues have been proposed as a key driver of Gd-IgA1 production (13). Supporting this concept, genome-wide association studies have also suggested genetic associations between IgAN and mucosal immune pathways including those related to microbial sensing and response (15, 16). These findings have motivated the introduction of tonsillectomy and enteric budesonide as therapeutic options (17, 18), and efforts are underway to utilize microbiota-modulating therapies including probiotics and fecal microbiota transplantation (19).

Nevertheless, the development of IgAN requires additional components, as described by the “multi-hit” model (14, 20). Mesangial cells actively contribute by binding IgA1 and driving proliferative signals (21). B cells play a pivotal role in producing Gd-IgA1 as well as autoantibodies against Gd-IgA1, although relatively little is known about their activation and potential interactions with the kidney glomeruli in IgAN (22). Unraveling the crosstalk among the gut microbiota, mucosal and systemic immune systems, and the kidney as a target organ is therefore critical for developing additional therapeutic strategies.

Data-driven, multi-omics approaches have the potential to yield novel insights into the complex pathophysiology of IgAN. Advances in high-throughput sequencing techniques have enabled comparative analyses of tonsillar or gut microbiome in patients with IgAN versus healthy or disease controls (21, 23–25). However, reported taxonomic differences varied among studies, and their functional relevance remains largely unclear. Spatial transcriptomics represent another key advancement, allowing for substructure-specific profiling of gene expression in elaborately organized structures like the nephron (26). We previously explored human IgAN-specific glomerular transcriptomic changes to uncover proinflammatory signals associated with mesangial proliferation preceding overt morphologic changes (27).

In this study, we performed 16S rRNA-based gut microbiome profiling integrated with Nanostring GeoMx-based glomerular spatial transcriptomics to characterize the molecular signature of IgAN. Comparisons with healthy controls and other glomerular disease patients enabled identification of specific changes in both the gut microbiome and the glomerular transcriptome. Specifically, IgAN showed gut microbial alterations in short-chain fatty acid (SCFA) metabolism, particularly acetate, along with glomerular transcriptional changes in SCFA transporters and G protein-coupled receptors (GPCRs). Together, these findings suggest a previously underappreciated axis of gut microbial metabolites and impaired renal sensing via GPCR downregulation, offering a potential channel for the gut-kidney interaction in IgAN.

## 2 Materials and methods

### 2.1 Ethics approval

This study was conducted in accordance with the Declaration of Helsinki, and ethical approval was granted by the Institutional Review Board (IRB) of Seoul National University Hospital (SNUH) for gut microbiome analysis (IRB No. 2205-104-1325) and spatial transcriptomic profiling (IRB No. 2205-085-1324). Written informed consent was obtained from all patients included in the study prior to collection of any samples or clinical information.

### 2.2 Participant cohorts and biospecimen acquisition

For gut microbial analysis, stool samples were sourced from the KOrea Renal biobank NEtwoRk System TOward Next-generation analysis (KORNERSTONE) repository (28). Biopsy-proven cases of IgAN and other common glomerular diseases, namely diabetic nephropathy (DN), minimal change disease (MCD), and membranous nephropathy (MN), were included. In addition, stool samples from consenting live kidney donors at SNUH were included as healthy controls. The following inclusion criteria were applied: (1) age ≥18 years, (2) estimated glomerular filtration rate (eGFR) ≥60 mL/min/1.73 m^2^ to minimize the potential confounding from uremic metabolites we previously observed (29), (3) no exposure to antibiotics or immunosuppressant treatment within one month of sample collection, (4) no history of bowel resection or inflammatory bowel disease.

For spatial transcriptomics analysis, IgAN samples were chosen from archived formalin-fixed paraffin-embedded (FFPE) slides, with biopsies performed between 2018 and 2022. Time-zero allograft biopsies after living donor kidney transplantation served as healthy controls, while DN, MCD, and MN samples archived between 2009 and 2021 were selected as disease controls. The selected participants had age 18–70 years, eGFR ≥30 mL/min/1.73 m^2^, and no immunosuppressant admission prior to biopsy to minimize the confounding from changes related to advanced aging, late-stage chronic kidney disease, or treatment decisions. Cases with fewer than 10 sampled glomeruli and non-DN cases with DN involvement on pathology were excluded.

### 2.3 Gut microbiome profiling

Stool DNA extraction and sequencing protocols were performed as described previously (29). Stool samples were collected on the day of kidney biopsy, immediately stored at -20 °C, then moved to -80 °C storage within 24 hours. DNA was extracted with the QIAamp Fast DNA Stool Mini Kit (Qiagen, Hilden, Germany). The V3-V4 hypervariable region of the 16S rRNA gene was amplified, followed by library preparation and sequencing through the Illumina MiSeq system according to the manufacturer’s protocol (Illumina, CA, USA).

For data analysis, raw sequence reads in FASTQ format were imported into the Quantitative Insights Into Microbial Ecology (QIIME2) microbiome analysis platform. The sequences were processed through DADA2 for filtering, trimming, and correction for low-quality reads and chimeric sequences (30). The denoised amplicon sequence variants were then annotated up to the genus level using the SILVA 138 database as reference (31). For diversity analysis, the samples were rarefied to a sampling depth of 10,000, and Shannon diversity indices and Bray-Curtis distances were computed through the QIIME2 q2-diversity plugin. Prediction of metagenomic functions was performed through Phylogenetic Investigation of Communities by Reconstruction of Unobserved States 2 (PICRUSt2) (32), and the predicted functions for each sample were expressed in terms of MetaCyc pathway abundances (33). Microbial genera and functional pathway abundances were compared between IgAN and each control group using ANOVA-like differential expression 2 (ALDEx2) (34), where false-discovery rates (FDR) < 0.05 were considered statistically significant.

### 2.4 Spatial transcriptomic profiling of glomeruli

Slide preparation and processing procedures for spatial transcriptomics profiling were described in detail previously (27). Briefly, 5 µm FFPE kidney biopsy sections underwent deparaffinization and epitope retrieval. A total of 18,677 target genes were labeled via *in situ* hybridization with the GeoMx Whole Transcriptome Atlas, which contains ultraviolet-photocleavable oligonucleotide identifiers. Three representative glomeruli per sample were selected as regions of interest. The oligonucleotide identifiers were collected, amplified through polymerase chain reactions, and sequenced on an Illumina NovaSeq 6000.

Data preprocessing and quality control were performed with the GeoMx Digital Spatial Profiler Data Analysis Suite (v2.4), removing low-performing genes, defined as those expressed in fewer than 50% of samples or below the limit of quantitation (LOQ). The LOQ was set as 2.0 standard deviations above the geometric mean of negative probes. The DESeq2 R package (v3.6.2), which employs negative binomial generalized linear models with median-of-ratios normalization, was used to identify differentially expressed genes (DEGs) between IgAN and each control group (35). Significant DEGs, defined as those with FDR < 0.10, were extracted for each comparison. Finally, the significant DEGs that show consistently high or low expression in IgAN across all comparisons were processed through the ToppGene Suite for Gene Ontology-based functional enrichment analysis, where a FDR < 0.05 was considered significant (36).

### 2.5 Serum and fecal acetate quantification by LC-MS

Acetate concentrations in serum and fecal samples were quantified using liquid chromatography–mass spectrometry (LC-MS) after derivatization with 3-nitrophenylhydrazine (3-NPH), as previously described (37). Fecal samples (~20 mg) were extracted in assay diluent buffer (water:acetonitrile, 1:1), centrifuged, and the supernatants were used for derivatization. For serum, 20 µL of sample was directly derivatized without prior extraction. Derivatized samples were incubated at 40 °C for 30 minutes and then diluted for analysis. LC-MS analysis was performed using a Q-Exactive Focus Orbitrap mass spectrometer (Thermo Fisher Scientific) coupled to a BEH C18 column (2.1 × 100 mm, 1.7 µm, Waters) under electrospray ionization in negative mode. Chromatographic separation was achieved using a water/acetonitrile gradient containing 0.1% formic acid at a flow rate of 0.35 mL/min. The mass spectrometer was operated in full scan mode (m/z 70–900), and acetate was identified and quantified by the retention time and m/z (194.1) of its derivatized form, which matched a derivatized acetate standard.

### 2.6 Statistical analysis and data visualization

All statistical analysis was performed in R (v4.4.0) except for gut microbial diversity analysis, which was performed on the QIIME2 platform using the q2-diversity plugin. For clinical characteristics, normality of continuous variables was assessed using histograms and skewness evaluations. For statistical comparisons between groups, Mann-Whitney U tests were used for two groups and Kruskal-Wallis was used for three or more groups. Linear associations between continuous variables were evaluated using the Pearson correlation coefficient. Permutational multivariate analysis of variance (PERMANOVA) with 999 permutations was used to evaluate differences in beta diversities among groups using the vegan R package (v2.7-1) (38). The Benjamini–Hochberg method was applied in calculations of false-discovery rates in cases of multiple testing. All boxplots and scatterplots were created with the ggpubr R package (v0.6.1) (39). Dotplot of the enriched Gene Ontology terms was visualized with the scToppR R package (v0.99.0) (40).

## 3 Results

### 3.1 Participant demographics and clinical profiles

A total of 336 participants were included in the gut microbiome analysis comprising 172 IgAN patients, 51 healthy donor controls, and 113 disease controls (**Table 1**). The IgAN patients had a mean age of 41.8, and 52% were female. The mean body mass index was 24.6 kg/m^2^ for IgAN, comparable to control groups, which ranged between 23.7 and 25.5 kg/m^2^. The mean eGFR ranged from 85.4 to 104.9 mL/min/1.73 m^2^ for each group.

**Table 1:** Baseline clinical characteristics of study participants subject to gut microbial profiling.

Characteristic	IgA nephropathy (N = 172)	Healthy donor (N = 51)	Diabetic nephropathy (N = 15)	Minimal change disease (N = 35)	Membranous nephropathy (N = 63)
Age (years)	41.8 ± 14.8	46.8 ± 12.2	47.1 ± 13.3	45.6 ± 18.0	55.8 ± 11.1
Female sex (N (%))	90 (52.3)	27 (52.9)	6 (40.0)	18 (51.4)	26 (41.3)
Body mass index (kg/m^2^)	24.6 ± 4.0	23.7 ± 2.8	25.5 ± 3.5	25.5 ± 3.6	25.1 ± 3.0
Body weight (kg)	67.2 ± 13.6	63.6 ± 10.3	72.3 ± 11.6	69.7 ± 12.8	67.6 ± 13.0
Systolic BP (mmHg)	125.2 ± 15.3	121.0 ± 13.4	126.0 ± 17.5	123.0 ± 13.8	126.7 ± 14.6
Diastolic BP (mmHg)	77.7 ± 11.0	76.5 ± 8.3	81.7 ± 13.3	77.5 ± 10.2	78.7 ± 9.3
Diabetes mellitus (N (%))	8 (4.7)	0 (0.0)	15 (100)	1 (2.9)	9 (14.3)
Hypertension (N (%))	63 (36.6)	8 (15.7)	12 (80.0)	7 (20.0)	29 (46.0)
eGFR (mL/min/1.73 m^2^)	96.4 ± 23.7	104.9 ± 12.1	85.4 ± 21.8	100.5 ± 20.7	98.7 ± 16.3
Hemoglobin (g/dL)	13.2 ± 1.8	14.0 ± 1.3	13.1 ± 2.1	14.5 ± 1.9	13.1 ± 1.5
Albumin (g/dL)	4.0 ± 0.5	4.4 ± 0.4	4.0 ± 0.6	2.5 ± 0.7	2.9 ± 0.7
Total cholesterol (mg/dL)	186 [161, 212]	186 [162, 218]	169 [148, 202]	385 [284, 514]	232 [182, 280]
Spot urine PCR (g/g)	0.97 [0.48, 1.75]	0.05 [0.04, 0.08]	3.40 [1.01, 3.92]	7.41 [4.34, 9.88]	4.51 [2.29, 6.66]
≥ 3.0 (N (%))	11 (6.4)	0 (0.0)	8 (53.3)	30 (85.7)	44 (69.8)
< 3.0 (N (%))	160 (93.0)	47 (92.2)	7 (46.7)	5 (14.3)	19 (30.2)
Not quantified (N (%))	1 (0.6)	4 (7.8)	0 (0.0)	0 (0.0)	0 (0.0)

BP, blood pressure; eGFR, estimated glomerular filtration rate; N, number; PCR, protein-to-creatinine ratio.

Parameters with normal distributions are shown as mean ± standard deviation, while other parameters are presented as median [interquartile range].

For spatial transcriptomic analysis, 8 biopsy-proven IgAN cases were subject to spatial transcriptomics profiling along with 10 healthy controls and 35 disease controls (**Table 2**). The IgAN patients had a mean age of 36.6, younger than control groups. They were more female, while control groups had a male majority. Lower eGFRs were observed among other disease controls, with the DN group having the lowest mean eGFR at 56.3 mL/min/1.73 m^2^.

**Table 2:** Baseline clinical characteristics of study participants subject to glomerular spatial transcriptomics profiling.

Characteristic	IgA nephropathy (N = 8)	Healthy donor (N = 10)	Diabetic nephropathy (N = 6)	Minimal change disease (N = 13)	Membranous nephropathy (N = 16)
Age (years)	36.6 ± 12.4	51.2 ± 8.6	56.0 ± 9.8	48.0 ± 12.4	52.1 ± 10.3
Female sex (N (%))	6 (75.0)	5 (50.0)	0 (0.0)	4 (30.8)	3 (18.8)
eGFR (mL/min/1.73 m^2^)	109.6 ± 20.3	89.2 ± 7.5	56.3 ± 24.4	75.7 ± 38.3	92.3 ± 23.5
≥ 90 (N (%))	7 (87.5)	4 (40.0)	1 (16.7)	6 (46.2)	11 (68.8)
≥ 60 and < 90 (N (%))	1 (12.5)	6 (60.0)	1 (16.7)	2 (15.4)	3 (18.8)
≥ 45 and < 60 (N (%))	0 (0)	0 (0)	1 (16.7)	2 (15.4)	1 (6.3)
≥ 30 and < 45 (N (%))	0 (0)	0 (0)	3 (50.0)	3 (23.1)	1 (6.3)
Hemoglobin (g/dL)	12.0 ± 1.3	14.5 ± 1.4	11.6 ± 2.0	13.2 ± 1.6	12.8 ± 1.2
Albumin (g/dL)	4.2 ± 0.4	4.0 ± 0.3	3.8 ± 0.7	2.1 ± 0.5	2.4 ± 0.7
Total cholesterol (mg/dL)	227 [205, 249]	201 [191, 213]	193 [177, 210]	360 [338, 440]	282 [216, 338]
Spot urine PCR (g/g)	1.03 [0.34, 1.97]	0 [0, 0]	2.73 [1.60, 3.07]	9.27 [6.86, 11.30]	7.36 [4.48, 9.03]
≥ 3.0 (N (%))	1 (12.5)	0 (0.0)	2 (33.3)	12 (92.3)	14 (87.5)
< 3.0 (N (%))	7 (87.5)	10 (100.0)	4 (66.7)	1 (7.7)	2 (12.5)

eGFR, estimated glomerular filtration rate; N, number; PCR, protein-to-creatinine ratio.

Parameters with normal distributions are shown as mean ± standard deviation, while other parameters are presented as median [interquartile range].

### 3.2 IgAN-specific functional enrichment of gut microbiota

**Figure 1:**
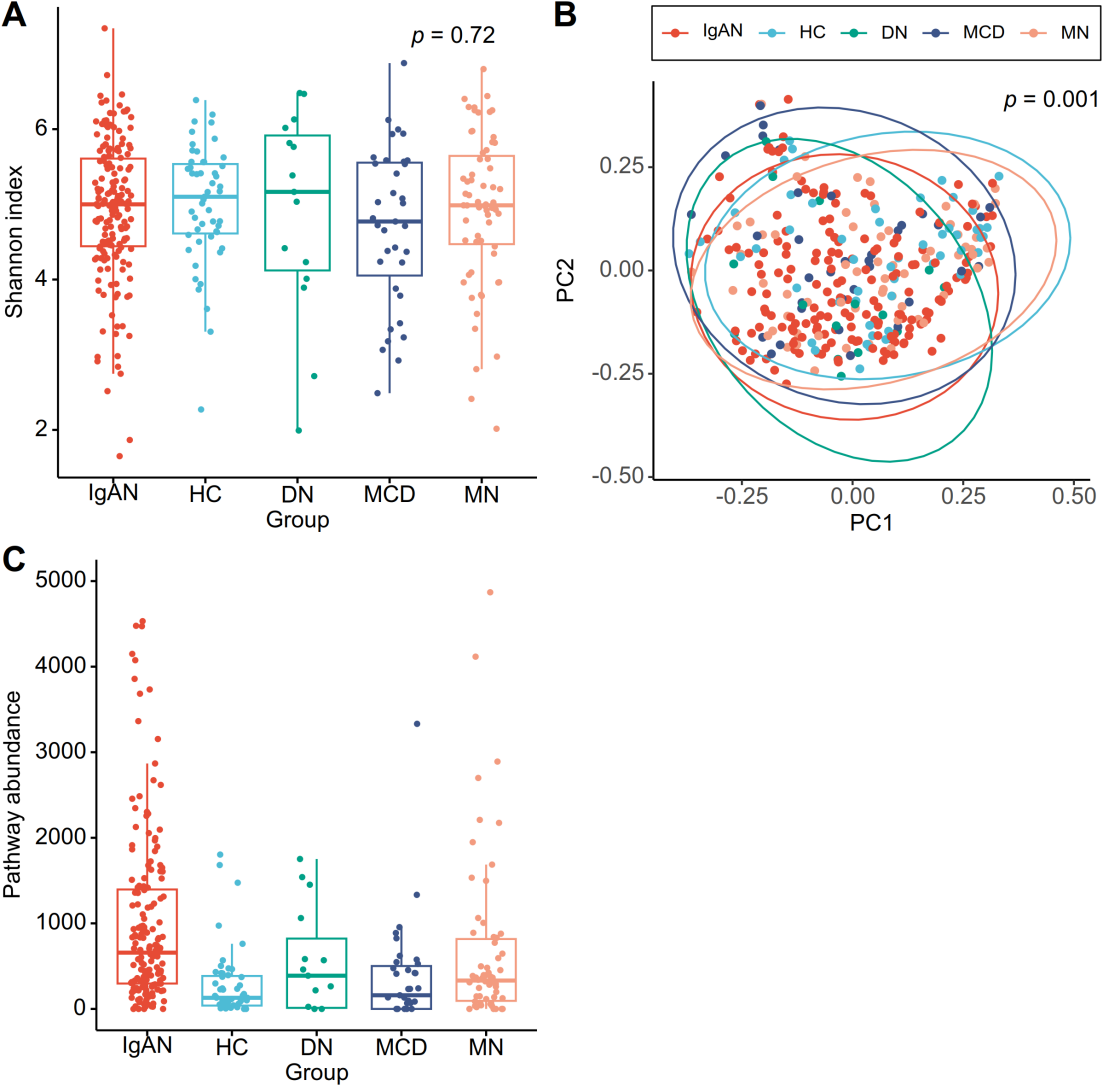
Characteristics of the gut microbiome in IgA nephropathy (IgAN). **(A)** Alpha diversity based on Shannon indices. The Kruskal-Wallis test was used to evaluate statistical significance. (HC: healthy controls, DN: diabetic nephropathy, MCD: minimal change disease, MN: membranous nephropathy) **(B)** Beta diversity based on Bray-Curtis dissimilarity indices represented as a principal coordinate analysis plot. Statistical significance was evaluated by permutational multivariate analysis of variance (PERMANOVA). (PC: principal coordinate) **(C)** Predicted abundances of the methanogenesis from acetate MetaCyc pathway grouped by diagnosis. Absolute pathways abundances were derived from PICRUSt2-predicted metagenome functions.

A total of 466 microbial genera and 332 predicted metagenomic functional pathways were identified. While alpha diversities did not differ among groups (**Figure 1A**), beta diversities were significantly differed among diagnostic groups (PERMANOVA, *p* = 0.001) (**Figure 1B**). IgAN microbiota composition was distinct from that of healthy controls (*p* = 0.001), MCD (*p* = 0.034), and MN (*p* = 0.013) but not from DN (*p* = 0.34). Significant differences between IgAN and healthy controls were consistently observed across different beta diversity metrics including Jaccard indices (*p* = 0.001), unweighted UniFrac (*p* = 0.006), and weighted UniFrac (*p* = 0.001), as were the differences among the five diagnostic groups (**Supplementary Figure 1**). Beta diversities based on the Bray-Curtis metric also significantly differed among groups (*p* < 0.001) when major covariates (age, sex, and eGFR) were included in the model.

In taxonomic differential abundance analysis, eight genera (*Bacteroides*, *Parabacteroides*, *Alistipes*, *Blautia*, *Romboutsia*, *Dorea*, *Lachnospira*, and *Butyricicoccus*) differed between IgAN and healthy controls, but none were consistently different from all disease controls (**Table 3**). As for functional analysis, Methanogenesis from Acetate (METH-ACETATE-PWY) was the only pathway significantly enriched in IgAN relative to both healthy controls and to each of the different glomerular disease controls, as visualized in a plot of the predicted pathway abundances (**Figure 1C**).

**Table 3:** Microbial genera with statistically significant differential abundance for IgA nephropathy compared to healthy and disease controls.

Family	Genus	HC		DN		MCD		MN	
		*P*-value	FDR	*P*-value	FDR	*P*-value	FDR	*P*-value	FDR
Lachnospiraceae	*Blautia*	5.45E-08	1.89E-05	0.185	0.868	3.07E-03	0.204	2.73E-04	0.0714
Bacteroidaceae	*Bacteroides*	2.11E-07	4.02E-05	0.550	0.941	0.0493	0.445	0.0176	0.453
Rikenellaceae	*Alistipes*	7.59E-06	8.53E-04	0.228	0.878	0.116	0.567	0.0304	0.517
Lachnospiraceae	*Lachnospira*	3.40E-05	2.79E-03	0.328	0.896	0.737	0.923	0.468	0.898
Tannerellaceae	*Parabacteroides*	1.22E-04	7.29E-03	0.889	0.981	0.511	0.851	0.331	0.874
Butyricicoccaceae	*Butyricicoccus*	3.37E-04	0.0150	0.867	0.977	0.0353	0.403	0.155	0.773
Peptostreptococcaceae	*Romboutsia*	3.89E-04	0.0162	8.07E-03	0.556	0.346	0.757	0.575	0.924
Lachnospiraceae	*Dorea*	6.51E-04	0.0243	0.856	0.977	0.0783	0.490	0.0944	0.690

DN, diabetic nephropathy, FDR, false-discovery rate, HC, healthy control, MCD, minimal change disease, MN, membranous nephropathy.

Statistical significance was determined by ALDEx2 with a false-discovery rate threshold of 0.05 by the Benjamini-Hochberg method.

### 3.3 Altered acetate metabolism in IgAN

**Figure 2:**
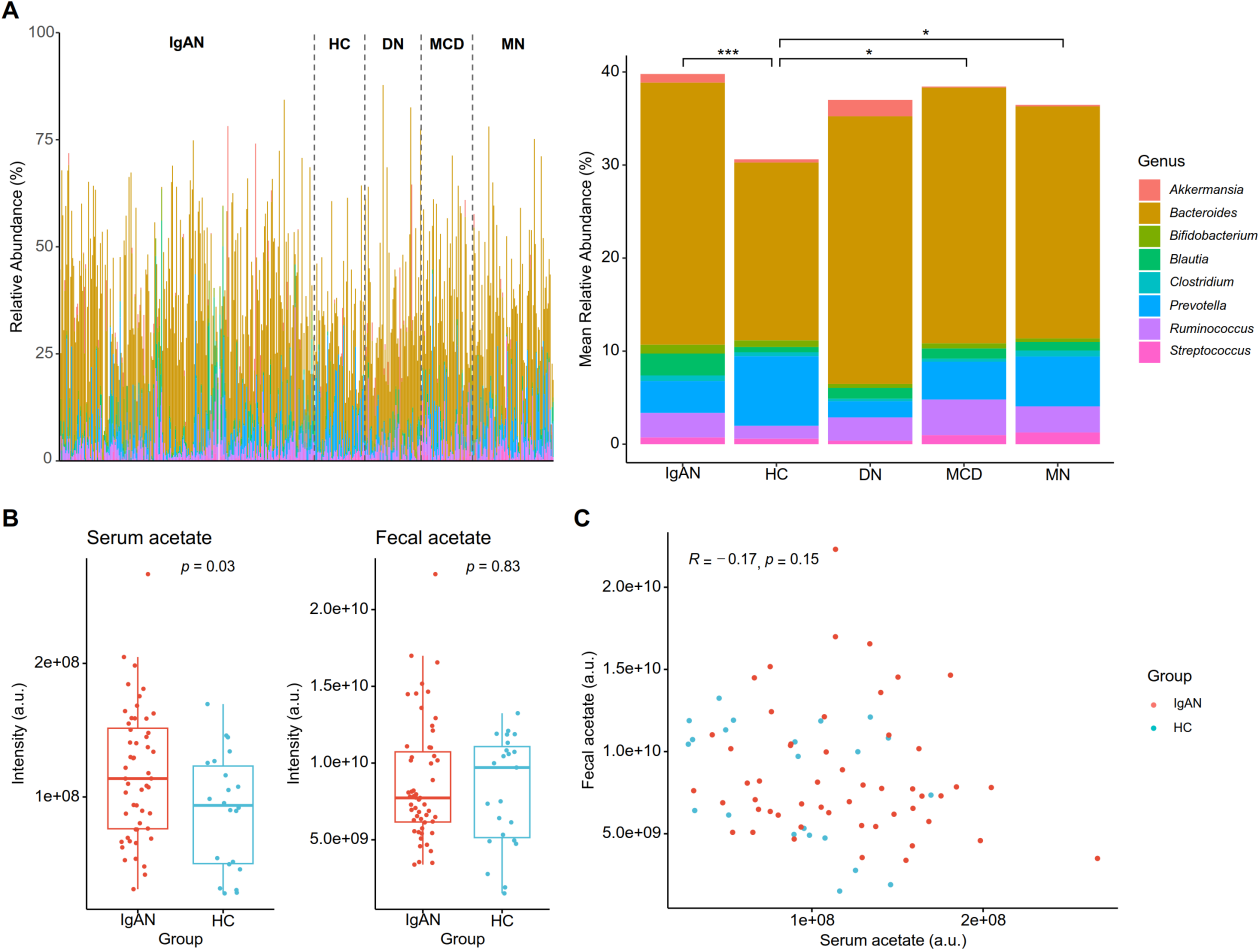
Acetate metabolism in IgA nephropathy (IgAN). **(A)** Relative abundances of major acetate-producing microbial taxa. The bar plot depicts the relative abundances of major acetate-producing genera for each sample, compiled into mean relative abundances for each diagnosis group. Pairwise Mann-Whitney U tests were performed for the combined mean relative abundances, and asterisks indicate statistically significant differences based on false discovery rates (FDR): *FDR < 0.05, ***FDR < 0.001. (HC: healthy controls, DN: diabetic nephropathy, MCD: minimal change disease, MN: membranous nephropathy) **(B)** Serum and fecal acetate levels in IgAN (N = 55) and HC (N = 23). Measurements from liquid chromatography–mass spectrometry are shown as intensities in arbitrary units (a.u.), and statistical significance was evaluated with Mann-Whitney U tests. **(C)** Correlation between measured serum and fecal acetate levels. Pearson correlation coefficient is shown, along with the corresponding *p*-value.

To assess the generation of acetate by the gut microbiota, we next compared relative acetate-producing potential based on the abundances of eight common gut microbial genera that contain species known to contribute to acetate production (**Figure 2A**). IgAN showed the highest mean total relative abundances of the eight genera at 39.8%, followed by disease controls at 37.3% (*p* = 0.037, FDR *=* 0.037) and healthy control at 30.6% (*p* < 0.001, FDR < 0.001). Among the 8 genera, *Bacteroides* and *Blautia* revealed higher abundance in IgAN than in healthy controls (**Table 3**). MCD (*p* = 0.008, FDR = 0.041) and MN (*p* = 0.014, FDR = 0.046) also showed elevated proportions of major acetate producers. Meanwhile, the acetotrophic methanogens recognized to possess the methanogenesis from acetate pathway, *Methanosarcina* and *Methanothrix*, were not detected in any sample. In addition to this, we found that IgAN patients had higher serum acetate levels than healthy controls (*p* = 0.03) but similar fecal acetate levels (**Figure 2B**). There was negligible correlation between serum and fecal acetate levels (*R* = -0.17, *p* = 0.15) (**Figure 2C**). The identified IgAN-associated gut microbial signatures, namely the relative acetate-producing potential and the methanogenesis from acetate pathway, did not show significant correlations with baseline clinical parameters or with serum and fecal acetate levels (**Supplementary Table 1**).

### 3.4 Glomerular spatial transcriptomics reveals altered signaling pathways in IgAN

**Figure 3:**
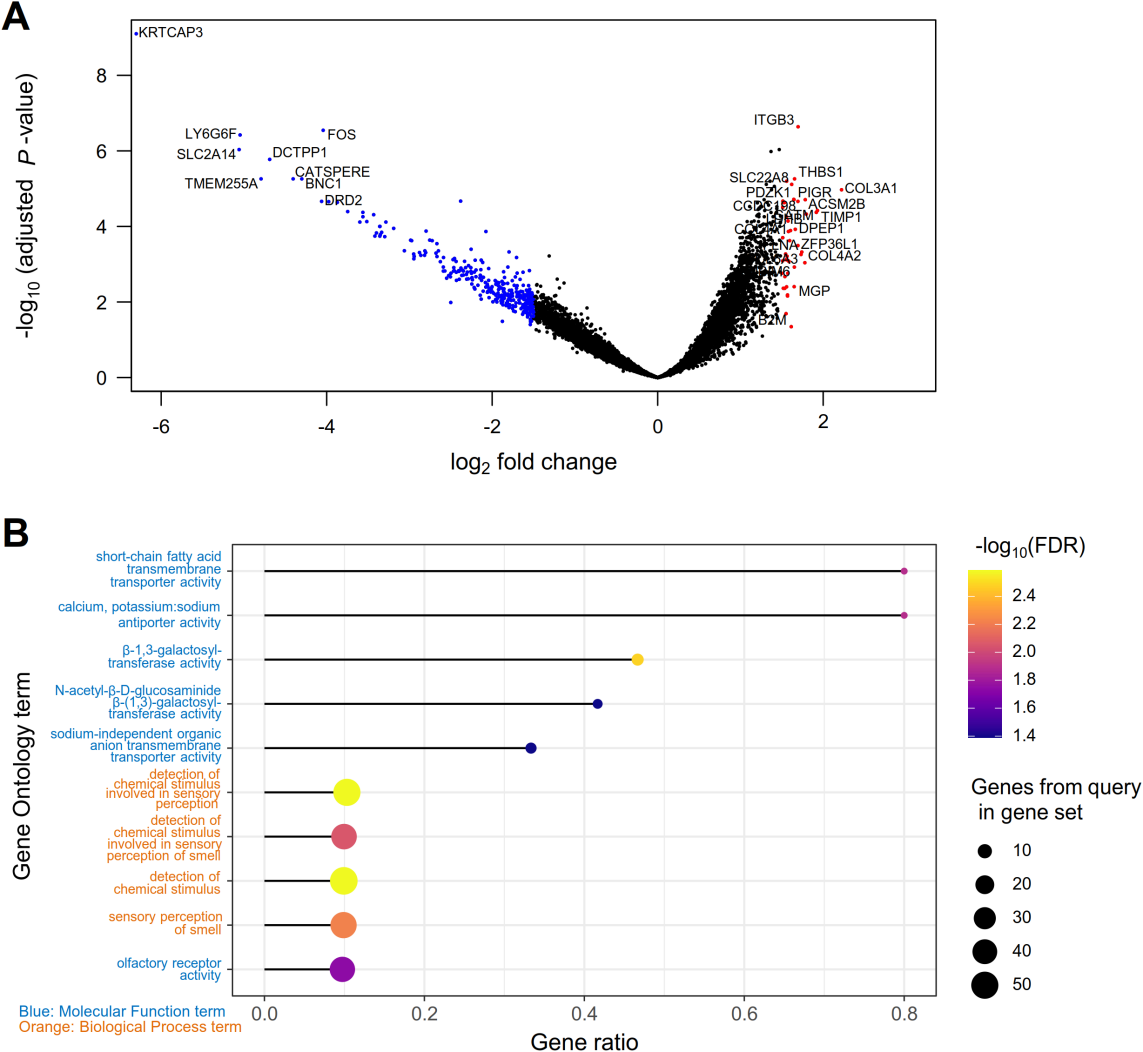
Differential gene expression analysis in the glomerular transcriptome of IgA nephropathy (IgAN). **(A)** Volcano plot of differentially expressed genes (DEGs) for the glomerulus of IgAN compared to healthy controls. Red and blue colors indicate DEGs with log_2_ fold change above 1.5 or below -1.5, respectively. **(B)** Gene Ontology (GO) terms significantly enriched among DEGs with decreased expression in IgAN. The x-axis, gene ratio, shows the ratio between the count of contributing DEGs and the total gene count for each GO term. Dot sizes show the absolute number of DEGs contributing to each GO term, while the dot colors correspond to the false discovery rate (FDR).

**Figure 4:**
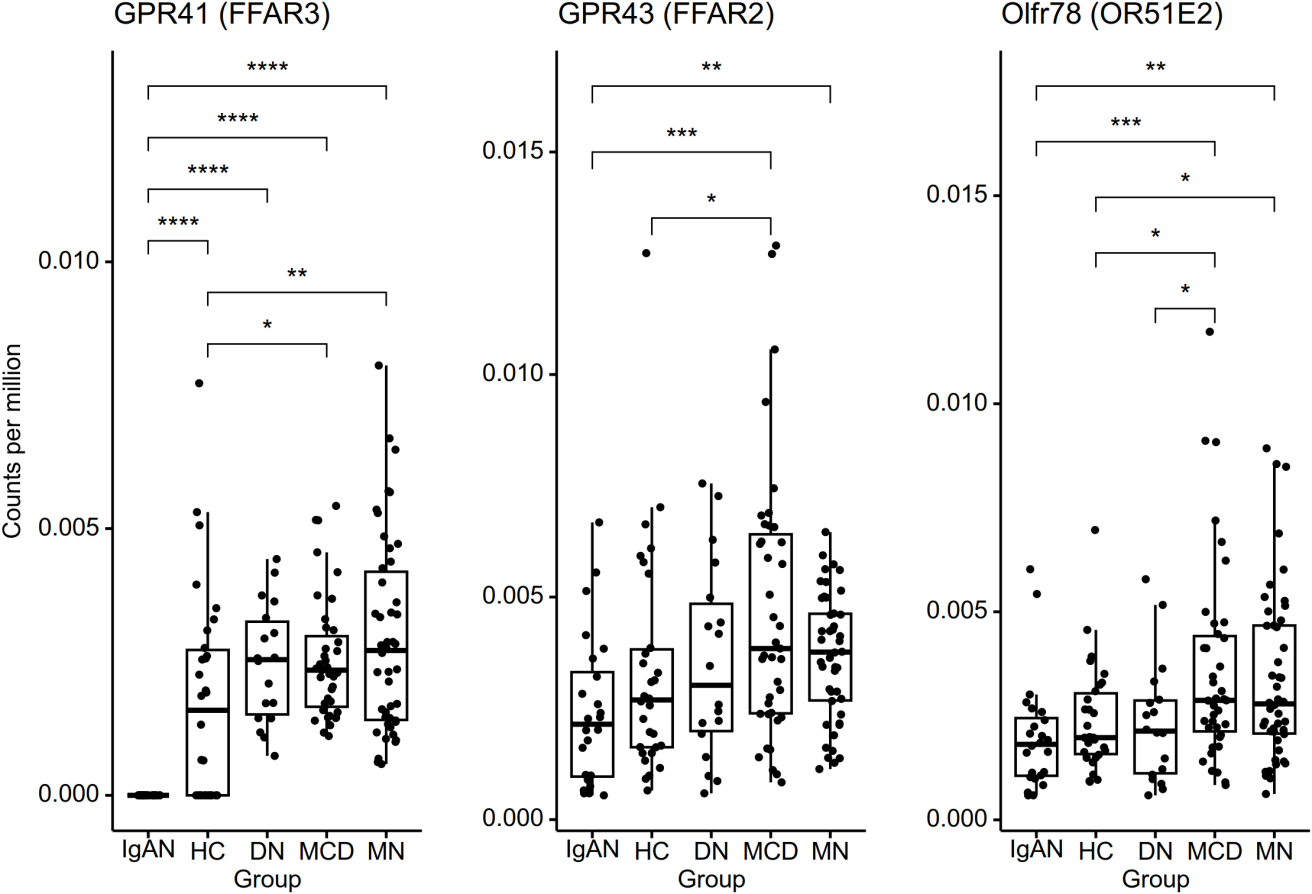
Glomerular expression of short-chain fatty acid-sensing G protein-coupled receptors. Statistically significant differences based on pairwise Mann-Whitney U tests are indicated with asterisks: *p < 0.05, **p < 0.01, ***p < 0.001, ***p < 0.0001.

Glomerular expression profiles of 17,834 genes were subject to analysis. Relative log expression plots showed no extreme outliers in count distributions (**Supplementary Figure 2A**), and compartment-specific gene expressions supported appropriate region selection (**Supplementary Figure 2B**). A total of 1,227 and 1,078 genes were consistently upregulated and downregulated, respectively, in IgAN relative to healthy controls and to each disease control group (**Figure 3A**, **Supplementary Tables 2**, **3**). The highest fold increase was found in *COL3A1* (collagen type III alpha 1 chain), a key component of the extracellular matrix. The highly expressed DEGs were associated with 1,209 Gene Ontology terms (**Supplementary Table 4**), where the top categories included cell adhesion, extracellular matrix organization, protein complex assembly, and mitochondrial function (**Table 4**). Meanwhile, the lowly expressed DEGs were enriched in 18 functional terms (**Table 4**, **Supplementary Table 5**). GPCR activity was the top enriched function, alongside terms pertaining to olfactory receptors, which belong to the GPCR superfamily. Beta-1,3-galactosyltransferase activity, involved in *O*-galactosylation of glycoproteins including immunoglobulins, also showed significant enrichment. Moreover, the majority of SCFA transmembrane transporter and calcium, potassium:sodium antiporter genes were downregulated in IgAN (**Figure 3B**). As for the acetate-sensing GPCRs, IgAN displayed undetectable *GPR41* (*FFAR3*) expression as well as lower *GPR43* (*FFAR2*) and *Olfr78* (*OR51E2*) expressions, although the latter was only significant relative to MCD and MN (**Figure 4**).

**Table 4:** Top gene ontologies for differentially expressed genes (DEGs) consistently upregulated or downregulated in IgA nephropathy.

Aspect	Terms increased in IgA nephropathy (up to 10 per aspect)	B-H FDR	Terms decreased in IgA nephropathy (up to 10 per aspect)	B-H FDR
Molecular function	oxidoreduction-driven active transmembrane transporter activity (GO:0015453)	6.27E-18	G protein-coupled receptor activity (GO:0004930)	3.74E-04
	electron transfer activity (GO:0009055)	6.27E-18	beta-1,3-galactosyltransferase activity (GO:0048531)	3.32E-03
	proton transmembrane transporter activity (GO:0015078)	7.04E-16	calcium, potassium:sodium antiporter activity (GO:0008273)	1.29E-02
	cell adhesion molecule binding (GO:0050839)	1.82E-15	short-chain fatty acid transmembrane transporter activity (GO:0015636)	1.29E-02
	NADH dehydrogenase (ubiquinone) activity (GO:0008137)	7.03E-13	transmembrane signaling receptor activity (GO:0004888)	1.75E-02
	cadherin binding (GO:0045296)	3.95E-12	olfactory receptor activity (GO:0004984)	1.82E-02
	primary active transmembrane activity (GO:0015399)	3.95E-12	sodium-independent organic anion transmembrane transporter activity (GO:0015347)	4.05E-02
	oxidoreductase activity, acting on NAD(P)H, quinone or similar compound as acceptor (GO:0016655)	4.79E-10	N-acetyl-beta-D-glucosaminide beta-(1,3)-galactosyltransferase activity (GO:0008499)	4.05E-02
	molecular adaptor activity (GO:0060090)	6.69E-09		
	protein-macromolecule adaptor activity (GO:0030674)	1.51E-08		
Biological process	protein-containing complex assembly (GO:0065003)	3.04E-19	detection of chemical stimulus involved in sensory perception (GO:0050907)	2.59E-03
	aerobic respiration (GO:0009060)	1.21E-18	detection of stimulus (GO:0051606)	2.59E-03
	oxidative phosphorylation (GO:0006119)	1.64E-17	nervous system process (GO:0050877)	2.59E-03
	cellular respiration (GO:0045333)	1.02E-16	detection of chemical stimulus (GO:0009593)	2.59E-03
	ATP biosynthetic process (GO:0006754)	1.17E-16	detection of stimulus involved in sensory perception (GO:0050906)	2.59E-03
	proton motive force-driven ATP synthesis (GO:0015986)	1.44E-16	G protein-coupled receptor signaling pathway (GO:0007186)	2.83E-03
	aerobic electron transport chain (GO:0019646)	2.31E-16	sensory perception of chemical stimulus (GO:0007606)	2.83E-03
	ribonucleoside triphosphate biosynthetic process (GO:0009201)	5.83E-16	sensory perception of smell (GO:0007608)	5.98E-03
	nucleoside triphosphate biosynthetic process (GO:0009142)	5.83E-16	sensory perception (GO:0007600)	5.98E-03
	purine ribonucleoside triphosphate biosynthetic process (GO:0009206)	5.83E-16	detection of chemical stimulus involved in sensory perception of smell (GO:0050911)	8.76E-03
Cellular component	mitochondrion (GO:0005739)	1.30E-22	None	
	mitochondrial envelope (GO:0005740)	1.20E-21		
	organelle envelope (GO:0031967)	8.34E-21		
	organelle inner membrane (GO:0019866)	1.31E-20		
	mitochondrial inner membrane (GO:0005743)	1.31E-20		
	catalytic complex (GO:1902494)	1.37E-20		
	mitochondrial membrane (GO:0031966)	2.10E-20		
	focal adhesion (GO:0005925)	8.61E-20		
	cell-substrate junction (GO:0030055)	1.20E-19		
	respiratory chain complex (GO:0098803)	2.15E-17		

B-H FDR, Benjamini–Hochberg false-discovery rate.

Up to 10 gene ontology terms with lowest false discovery rates for each aspect are shown.

## 4 Discussion

In this study, we integrated gut microbiome profiling and glomerular spatial transcriptomics to characterize the microbial and transcriptomic changes in IgAN that potentially modulate the gut-kidney axis. The gut microbiome in IgAN showed an enrichment of major acetate-producing taxa and the methanogenesis from acetate pathway, accompanied by elevated serum acetate levels. At the same time, the glomerular transcriptome of IgAN demonstrated a downregulation of SCFA transporters and SCFA-sensing GPCRs, suggesting a mismatch between systemic metabolite availability and kidney sensing capacity.

Mounting evidence suggests acetate plays a key role in regulating immunity and inflammation, including in kidney diseases. Primarily generated through gut bacterial fermentation, acetate is the predominant SCFA, constituting 60-75% of total gut SCFAs (41, 42) and over 90% of those in serum (43). As both an energy source and a signaling molecule, it supports maintenance of the intestinal epithelial barrier while suppressing proinflammatory mediators (41). *In vivo*, acetate prevented acute kidney injury in an ischemia-reperfusion injury model and reduced kidney fibrosis in an unilateral ureteral obstruction model (44, 45), and it also attenuated clinical manifestations in a mouse model of IgAN (46). Hence, acetate may have renoprotective effects specific to IgAN as well as across kidney disease more broadly.

Our gut microbial analysis suggests that acetate production and consumption in the gut are both modulated in IgAN. Functional analysis indicated an increase in predicted enzymatic activities attributable to acetate consumption by methanogens. While methanogens have been associated with several diseases including inflammatory bowel disease (47), research on their role in the human gut is relatively sparse. Since methanogens were not identified in our taxonomic annotations, possibly because archaea comprise only 0.1% of reads in non-targeted sequencing (48), the observed metabolic activities may also reflect contributions from other acetate utilizers. As for acetate production, a previous study reported significantly decreased fecal SCFA levels in IgAN, with correlations to gut microbiota composition and clinical parameters, suggesting that disruption of SCFA levels could be relevant to IgAN (49). However, we did not observe differences in fecal acetate levels, and the relative increase in major acetate-producing genera in IgAN suggests that acetate production may not be compromised in IgAN. Our results are therefore most consistent with a simultaneous increase in the consumption and production of gut acetate in IgAN.

In addition, the glomerular transcriptome of IgAN shows potential evidence of altered acetate signaling in the kidney. Acetate in circulation can signal across diverse tissues through specific GPCRs (GPR41, GPR43, Olfr78) (50), which were downregulated in the glomerular transcriptome of IgAN: *GPR41* was undetectable, and *GPR43* and *Olfr78* had significantly lower expression compared to MCD and MN. As *GPR41* expression was also undetected in some of the healthy controls, the observed lack of *GPR41* expression in IgAN likely reflects its low baseline expression below the detection threshold. We also observed a downregulation of SCFA transmembrane transporters, namely *SMCT* (*SLC5A8*), *OAT4* (*SLC22A9*), *OAT5* (*SLC22A10*), and *UST5* (*SLC22A25*). These transporters are mainly associated with renal tubules rather than the glomerulus, but they are also part of the organic anion transporter family, whose members can mediate interorgan communication by modulating metabolite levels (51). The downregulation of olfactory receptor activity in IgAN could also reflect decreased SCFA signaling, as some renal olfactory receptors have physiological functions. Olfr78, as a prime example, localizes to the juxtaglomerular apparatus and modulates renin homeostasis through acetate and propionate signaling (52). Other downregulated orphan olfactory receptors may similarly localize to the glomerulus and contribute to acetate signaling. While these findings may indicate compensatory responses to elevated acetate levels, negative feedback is not universal. Indeed, a recent study showed that acetate exposure upregulated, rather than downregulated, *GPR43* expression in kidney tubular cells (53). Hence, the glomerular downregulation of SCFA-related genes in IgAN may reflect pathologic suppression of acetate signaling, which would leave the kidney vulnerable to inflammation and oxidative stress.

Collectively, our analyses present a nuanced picture of acetate biology in IgAN that involves potential changes from synthesis in the gut to signaling in the glomerulus. While serum acetate levels were elevated in IgAN, this may be a reactive change rather than a pathological mechanism. Elevations in circulating acetate levels have been observed in acute stress conditions such as bacterial infections, where acetate facilitates memory T cell activity (54). Thus, the absolute increase in serum acetate levels in IgAN may not meet physiologic needs in acute inflammatory conditions, exacerbated by the downregulation of potential SCFA sensors in the glomerulus. The lack of correlation between fecal and serum acetate levels further demonstrates the complexity of acetate dynamics in IgAN. Previous studies in healthy individuals also did not report clear correlations between the two measurements despite rapid absorption of fecal acetate (43, 55).

In the kidney, acetate signaling may modulate diverse molecular pathways, with potential links to pathophysiologic processes underlying glomerular diseases. One possibly relevant mechanism is the nuclear transcription factor kappa B (NF-κB) pathway, a key regulator of inflammation across glomerular diseases and CKD (56–58). SCFA-activated GPR43 inhibits the NF-κB pathway and oxidative stress in DN, suggesting protective actions of acetate (59). At the same time, SCFAs can also amplify signaling from aryl hydrocarbon receptors, which can stimulate NF-κB-driven inflammation and suppress Nrf2 antioxidant pathways (60, 61). Acetate may also affect the kidney by modulating the renin-angiotensin system. Given the increased circulating acetate levels in IgAN, Olfr78 in the juxtaglomerular apparatus could be highly activated, promoting renin secretion and leading to glomerular hypertension. Indeed, a study of early DN identified elevated serum acetate levels in a rat model and suggested that excess acetate may promote kidney injury through the renin-angiotensin system (62). Also downstream of the renin-angiotensin system is the Wnt/β-catenin pathway, whose persistent activation can lead to kidney fibrosis in glomerular diseases and CKD (63, 64). Conversely, the counterbalancing effects of GPR41 on blood pressure, together with the downregulation of Olfr78, could blunt or even reverse these effects on the regulation of renin (65). As such, renal acetate signaling in IgAN may affect multiple molecular mechanisms, whose contributions to pathophysiology are likely context dependent.

Beyond its effects on glomerular inflammation, acetate may also contribute to the pathophysiology of IgAN through signaling in other cell populations. Although not directly addressed by the current analysis, earlier *in vivo* studies have suggested that acetate can regulate gut mucosal immune cells. Specifically, acetate supplementation induced gut IgA production and regulated IgA reactivity to microbes through stimulation of dendritic cells, which promote IgA class switching and gut homing in B cells (66). In certain contexts, acetate can also induce dendritic cells to produce B-cell activating factor (BAFF) (66, 67), an emerging therapeutic target whose levels are elevated in IgAN (14). In addition, acetate stimulates CD4^+^CCR6^+^ T follicular helper-like cells by promoting CCL20 production in colonic epithelial cells, regulating T cell-dependent IgA production in germinal centers by modulating Toll-like receptor (TLR) signaling (68). This may also be relevant to IgAN pathogenesis, as a recent study found that transplanting gut microbiota from IgAN patients into mice activates TLR4 signaling and induces the disease phenotype, while TLR4 inhibition suppresses Gd-IgA1 production (69). In all, acetate can modulate IgA biology through its interactions with gut immune cells, such as dendritic cells and helper T cells, which may have potential relevance to the pathogenesis of IgAN.

Recent studies associate gut dysbiosis and metabolite dysregulation with kidney injury across glomerular diseases. Uremic toxin generation and impaired SCFA production, along with intestinal barrier dysfunction, have been identified as major pathways whereby gut dysbiosis contribute to CKD (70). The gut microbiome in MN shows reductions in *Lactobacillus*, whose tryptophan-derived metabolites can attenuate kidney damage by inhibition of aryl hydrocarbon receptor signaling (71). Significant gut dysbiosis was also observed in MCD that includes decreased abundance of the butyrate producer *Faecalibacterium* (72). As for DN, the gut microbiome is associated with multiple potentially pathogenic mechanisms including increased endotoxin levels, decreased SCFA levels, and dysregulated bile acid metabolism (73). As such, SCFAs are associated with multiple kidney diseases, typically showing reduced production, but their mechanistic roles remain incompletely defined.

SCFAs, in particular acetate, may therefore play a disease-specific role in IgAN. Our analysis suggests an overall increased activity of acetate metabolism in the gut, where it can boost IgA production and reactivity as previously discussed. At the same time, downregulation of genes responsible for SCFA transport and sensing in the glomerulus would limit acetate uptake and signaling, which can contribute to glomerular injury through impaired regulation of anti-inflammatory pathways, disruption of renin signaling, and promotion of local metabolic stress. The observed elevation in serum acetate may represent both a systemic response to inflammation and a compensatory adaptation to defective acetate signaling in the kidney.

Human studies demonstrate the clinical relevance of gut dysbiosis in IgAN, but the contribution of SCFAs such as acetate remains to be established. Gut microbiota profile could predict treatment response in IgAN (74), and a pilot trial suggested fecal microbiota transplantation (FMT) may serve as an adjunctive treatment (75). Nonetheless, despite several reports of decreased serum and fecal acetate levels in IgAN and in broader CKD (49, 76), SCFAs were not among the intestinal metabolites altered by FMT in IgAN in the pilot study. Adding to this complexity, our glomerular transcriptomic data indicated that acetate signaling in the kidney may depend on systemic availability as well as renal sensing capacity, highlighting the need to assess associated receptor functions when interpreting SCFA biology in kidney disease.

The current study has several limitations. First, the 16S rRNA data had limited resolution. Taxonomic annotation was restricted to the genus level, and certain taxa may not have been detected, particularly archaeal species for which the primer designs and taxonomic databases are less optimized. Functional annotation was also performed indirectly through PICRUSt2, which first derives the predicted metagenome from the 16S data and then algorithmically infers pathway composition. Further validation through metagenomic sequencing and functional assays is needed to verify the observed genus- and pathway-level associations. Second, due to the relatively low number of reads in our spatial transcriptomics data, we could not reliably localize gene expression patterns within the glomerulus, and some genes with low expression levels may have not been detected, as was likely the case for *GPR41*. Third, while efforts were made to select representative cases, our study may not reflect the entire spectrum of IgAN. The cases included in this study are all relatively young Koreans, which may not fully capture the nature of the disease in other ethnicities, especially given the known differences in clinical severity and gender distributions in Asian-Pacific populations (14). The low number of samples subject to glomerular transcriptomics also limits the generalizability of our findings. Finally, because this is a data-driven, observational study, the contributions of metabolites, glomerular genes, and gut microbial functions to IgAN pathophysiology remain to be established by experimental studies. Other SCFAs may also signal through the involved GPCRs and contribute to the postulated mechanisms, and additional confounding factors, such as dietary patterns, could influence gut microbial composition as well as SCFA levels. There may also be important transcriptional and microbial characteristics not captured by our analysis due to the limited sample size and the study design, which compared IgAN with four different control groups separately to minimize false positive results.

In conclusion, we have identified elevations in gut microbial acetate production and consumption activities as well as glomerular downregulation of GPCR, SCFA transport, and galactosylation functions in IgAN. Our results suggest that acetate may contribute to the pathophysiology of IgAN by mediating the gut-kidney interaction through GPCR signaling.

## Data Availability

The data presented in the study are deposited in the NCBI SRA repository, accession number PRJNA1399494.
