# Supplementary material for: Altered acetate metabolism and signaling in IgA nephropathy: an integrated gut microbiome and glomerular spatial transcriptome analysis
Source: Front Immunol. 2026 Jan 14;16:1665585. doi: 10.3389/fimmu.2025.1665585 (PMC12847407; doi:10.3389/fimmu.2025.1665585)

Supplementary Material

1 Supplementary Tables

Supplementary Table S1. Correlation analysis between consistently altered gut microbial signatures in IgA nephropathy and clinical parameters including serum and fecal acetic acid levels. Pearson's correlation coefficients and associated *p*-values are shown.

	**Relative abundance of acetate-producing taxa**		**Methanogenesis from acetate pathway**	
**Clinical parameter**	***R***	***p*-value**	***R***	***p*-value**
Age (years)	-0.052	0.50	-0.050	0.36
Body mass index (kg/m^2)	-0.038	0.62	0.054	0.32
Systolic BP (mmHg)	-0.118	0.12	0.053	0.34
Diastolic BP (mmHg)	-0.038	0.62	0.054	0.32
eGFR (mL/min/1.73 m^2)	0.119	0.12	-0.013	0.82
Hemoglobin (g/dL)	0.051	0.51	-0.023	0.67
Albumin (g/dL)	0.047	0.54	-0.007	0.90
Total cholesterol (mg/dL)	-0.0002	1.00	-0.057	0.33
Spot urine PCR (g/g)	0.029	0.71	-0.053	0.34
Serum acetate (a.u.)	0.110	0.35	0.120	0.31
Fecal acetate (a.u.)	-0.046	0.69	0.179	0.12

BP: blood pressure, eGFR: estimated glomerular filtration rate, a.u.: arbitrary units

(***Table S2-S5 are provided as a single Microsoft Excel file.***)

Supplementary Table S2. Differentially expressed genes with significantly high expressions in the glomerulus of IgAN relative to each control group. The genes are sorted from highest to lowest absolute fold changes in the IgAN versus healthy control comparison. Each column shows the mean of normalized counts across all samples in the comparison (baseMean), log2 fold change (log2FC), log fold change standard error (lfcSE), Wald test statistic (stat), *p*-value (pvalue), and Benjamini–Hochberg false-discovery rate as adjusted *p*-value (padj).

Supplementary Table S3. Differentially expressed genes with significantly low expressions in the glomerulus of IgAN relative to each control group. The genes are sorted from highest to lowest absolute fold changes in the IgAN versus healthy control comparison. Each column shows the mean of normalized counts across all samples in the comparison (baseMean), log2 fold change (log2FC), log fold change standard error (lfcSE), Wald test statistic (stat), *p*-value (pvalue), and Benjamini–Hochberg false-discovery rate as adjusted *p*-value (padj).

Supplementary Table S4. Functional enrichment analysis based on Gene Ontology (GO) for differentially expressed genes exhibiting consistently high expression in IgAN. GO terms with Benjamini–Hochberg false-discovery rate (FDR B&H) <0.05 are displayed, and entries are sorted by *p*-value for each GO category.

Supplementary Table S5. Functional enrichment analysis based on Gene Ontology (GO) for differentially expressed genes exhibiting consistently low expression in IgAN. GO terms with Benjamini–Hochberg false-discovery rate (FDR B&H) <0.05 are displayed, and entries are sorted by *p*-value for each GO category.

2 Supplementary Figures

Supplementary Figure 1. Principal coordinate analyses (PCoA) the gut microbiome based on alternate beta diversity metrics. **(A)** Unweighted UniFrac **(B)** Weighted UniFrac **(C)** Jaccard distance. Statistical significance was evaluated by permutational multivariate analysis of variance (PERMANOVA). (DN: diabetic nephropathy, HC: healthy controls, IgAN: IgA nephropathy, MCD: minimal change disease, MN: membranous nephropathy)

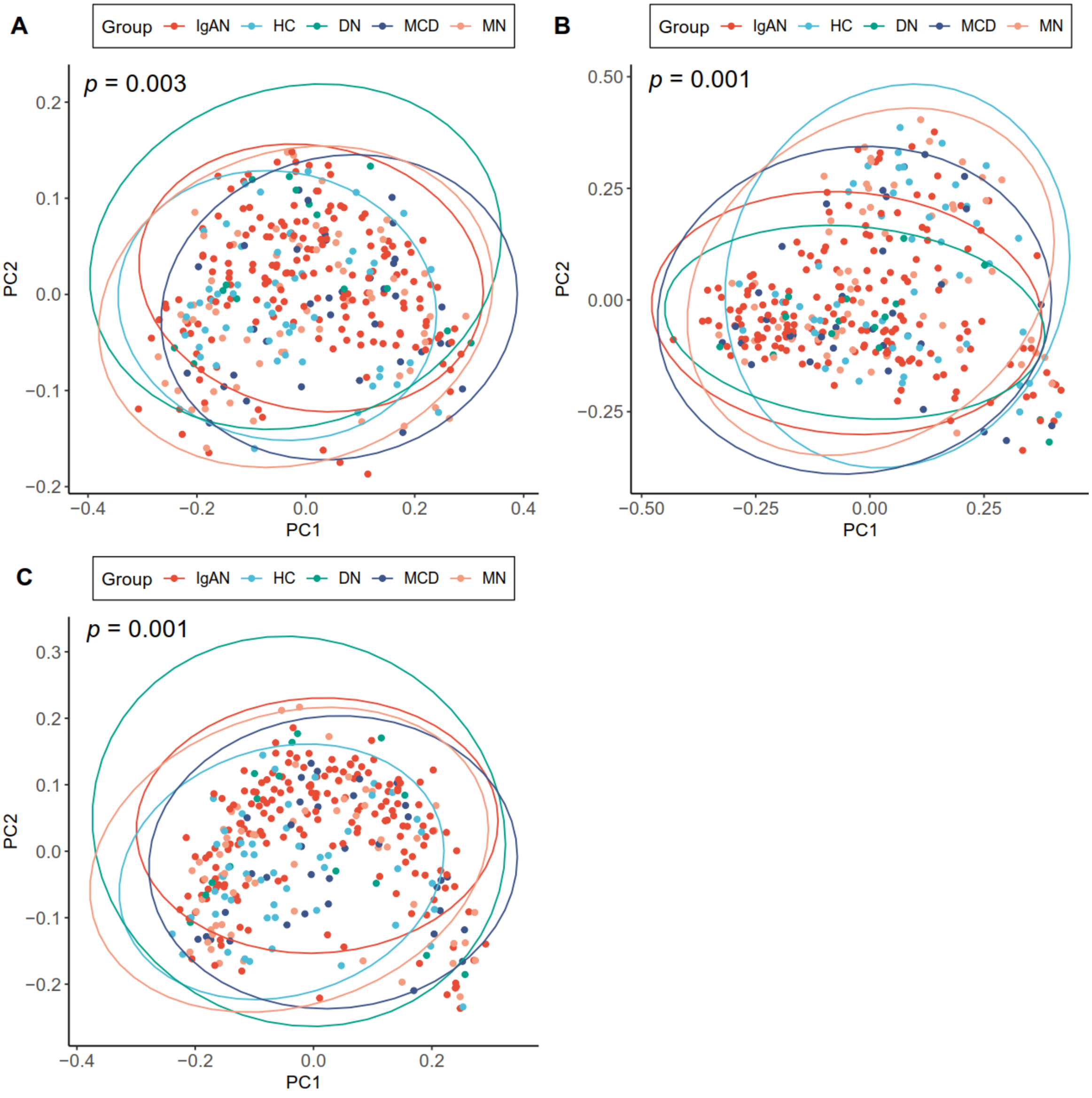

Supplementary Figure 2. Evaluation of the spatial transcriptomics profiling. **(A)** Relative log expression plot of read count distributions for each sample, sorted by diagnosis. **(B)** Relative expression levels of genes specific to glomerular (podocyte markers *PODXL* and *NPHS2*, endothelial cell marker *PECAM1*) and tubulointerstitial (*AQP2* for collecting ducts, *SLC12A3* for distal convoluted tubules, *LRP2* for proximal tubules) substructures.

(A)

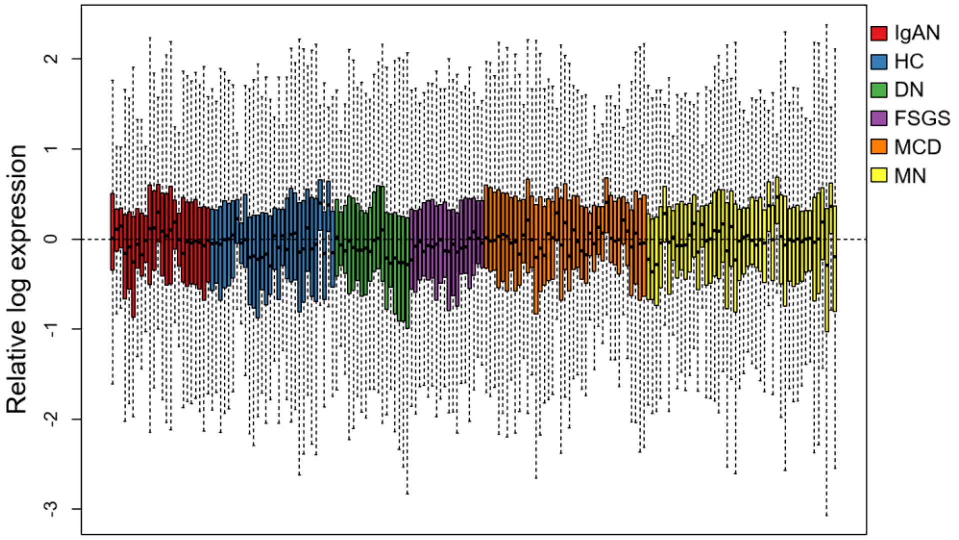

(B)

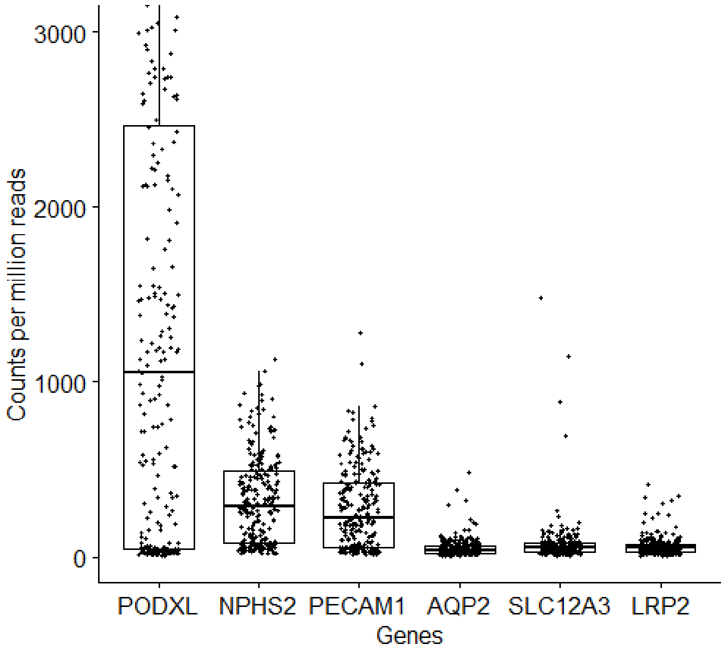

Supplement: Supplementary file 1 [file DataSheet1.pdf]
